# Patients’ attitudes towards privacy in a Nepalese public hospital: a cross-sectional survey

**DOI:** 10.1186/1756-0500-6-31

**Published:** 2013-01-29

**Authors:** Malcolm Moore, Ritesh Chaudhary

**Affiliations:** 1 Broken Hill Department of Rural Health, University of Sydney, PO Box 457, Broken Hill, NSW 2880, Australia; 2 BP Koirala Institute of Health Sciences, Dharan, Nepal

**Keywords:** Medical consultations, Nepal, Privacy

## Abstract

**Background:**

Many people in western countries assume that privacy and confidentiality are features of most medical consultations. However, in many developing countries consultations take place in a public setting where privacy is extremely limited. This is often said to be culturally acceptable but there is little research to determine if this is true. This research sought to determine the attitudes of patients in eastern Nepal towards privacy in consultations. A structured survey was administered to a sample of patients attending an outpatients department in eastern Nepal. It asked patients about their attitudes towards physical privacy and confidentiality of information.

**Findings:**

The majority of patients (58%) stated that they were not comfortable having other patients in the same room. A similar percentage (53%) did not want other patients to know their medical information but more patients were happy for nurses and other health staff to know (81%). Females and younger patients were more concerned to have privacy.

**Conclusion:**

The results challenge the conventional beliefs about patients’ privacy concerns in Nepal. They suggest that consideration should be given to re-organising existing outpatient facilities and planning future facilities to enable more privacy. The study has implications for other countries where similar conditions prevail. There is a need for more comprehensive research exploring this issue.

## Findings

### Background and research question

Most people in western countries assume that privacy and confidentiality are part of normal medical consultations. The confidentiality of the doctor-patient relationship dates back to antiquity. The Hippocratic Oath states, “What I see or hear in the course of the treatment in regard to the life of men, which on no account one must spread abroad, I will keep to myself holding such things shameful to be spoken about”. Privacy is a broader term including physical privacy, informational privacy, protection of personal identity and the ability to make choices without interference [1].

These things can be difficult to achieve in western settings and may involve complex judgements [2,3]. The knowledge of medical staff about principles of confidentiality can be lacking [4] and the expectations of patients very high [5]. In non-western settings expectations can be very different and the difficulties much greater. Many developing country consultations are conducted with several doctors in the same room, often at the same desk. There may be medical students as well, separately seeing patients. Patients each have one or two attendants and ancillary staff walk in and out freely. Privacy might mean occasionally pulling a screen around the examination bed. This scenario is usually found in medical practice in Nepal. It is partly a result of high patient numbers and limited manpower and facilities. However, anecdotally, many Nepalese doctors say that it is a cultural issue as well: information is shared and patients want the support of friends and family at every step. This emphasis on the group is often taken to indicate that people don’t want or expect privacy.

There is little research evidence about expectations of privacy in developing countries. Most western evidence was gathered decades ago, probably because expectations of privacy are now assumed. It also focuses on younger patients, who are found to be sensitive to issues of confidentiality. Most adolescents consult a GP more than once a year [6] but Ford et al. [7] found that this fell significantly if it was related to pregnancy, HIV, or substance misuse. Around 25% would forego health care if they had concerns about confidentiality. Finkenauer et al. [8] suggests that young women are unlikely to discuss sexual behavior with a doctor if they are not sure that their consultations will be confidential. Elsewhere, adolescents have been found to consider confidentiality a high priority [9-13]. Whiddet et al. [14] studied primary-care patients in New Zealand to investigate their attitudes toward sharing medical information. They found three factors that influenced attitudes: the identity of the recipient (e.g. health professional vs government body); the level of anonymity; and the type of information (e.g. very personal details).

Some relevant research has been done in non-western settings. A study in Egypt showed that one third of patients interviewed in a hospital outpatients clinic thought the level of privacy in the consultation room was unsatisfactory [15]. Bhatia and Cleland [16] in Karnataka State, India, found that there was significantly less privacy in public compared to private medical settings. In family practice clinics at the Aga Khan University in Pakistan [17], patients recorded objections to the presence of medical and nursing students and other observers on the basis of a reduction in privacy. There were also concerns over privacy from diabetic patients in Oman [18], and hospital patients in Lahore [19], particularly in the public system.

The literature supports the contention that privacy and confidentiality are important to patients. It also shows that these concerns are not only found in western settings. In Nepal some medical schools are beginning to emphasise principles of good communication and patient-centred care, often assuming physical privacy in the consultation. The authors of this paper worked and studied together in such a medical school for three years. This research was prompted by the tension between what was being taught – based on western curricula – and the reality of consulting in a busy Nepalese hospital where privacy may not be offered. The research question arose: what expectations of privacy do Nepalese patients have in this hospital? This question was broken down into questions about physical and informational privacy which queried the assumption that the patients were happy with the current situation.

## Methods

The study was performed in the general outpatients department (GOPD) of B.P. Koirala Institute of Health Sciences (BPKIHS) in eastern Nepal. This is a 700-bed teaching hospital with a GOPD seeing around 100 patients a day. A cross-sectional study was done using a structured survey instrument in Nepalese, containing 13 items. (Appendix) It asked patients about their attitudes towards physical privacy and confidentiality of information using mostly closed questions, with the opportunity for comments. This survey was piloted by the Nepalese researcher (RC), a doctor working in GOPD, and refined for use with patients from the target population. The population was all patients attending GOPD. A convenience sample of 100 individuals was chosen. This sample consisted of all patients who consulted the researcher during his rostered hours in the department and consented to inclusion. His weekly morning clinic usually fell on the same day but was subject to variation. After explaining the study and receiving oral consent, the researcher administered the survey. This was done orally due to the low literacy of the population. Where patients were less than 18 years of age their caregiver was approached for inclusion in the study. Interviews continued over a three-month period in 2010 until the arbitrary target of 100 participants was reached. Ethics approval for the study was given by the Research and Thesis Committee at BPKIHS.

Most of the data were quantitative. SPSS software 17.0 using chi-square test for difference in proportions was used for analysis. There was a small amount of qualitative data arising from the opportunity given to patients for comment on several questions. These data were analysed using an iterative process of thematic analysis: initially by each researcher individually and then together until agreement was reached.

## Results

100 patients were enrolled in the study, 2 patients declining to participate. There were 59 females and 41 males. 38 patients were housewives, 19 farmers, 18 students and the remainder had diverse occupations including teachers, labourers and shopkeepers or were unemployed. The age and educational demographic data are shown in Table 1.

**Table 1:** Age and educational level of participants, n=100

**Age**	**Number**
16-25	41
26-35	33
<35	26
**Educational level**	**Number**
No schooling	23
Primary (1–5)	14
High school (6–10)	43
Intermediate ( −12)	14
Bachelor degree	6

Patients were usually accompanied by one or two attendants (mean 1.1), all of whom came into the consultation. 75% of patients stated that they preferred all of their attendants to be present.

There were 2 questions directly related to the number of consultations occurring in each room (Table 2). These questions were separated in the patient survey.

**Table 2:** Number of consultations per room preferred by participants, n=100

**Question**	**Yes**	**No**
Q 4. Are you comfortable with having other patients in the same room as you, consulting with other doctors?	42	58
Q 11. Would you prefer if there was only one doctor in each consulting room?	56	44

Several questions were asked about the confidentiality of medical information in the consulting room as shown in Table 3. Patients were much more comfortable with nurses and helpers knowing their information (81%) than their attendants (55%) or other patients (53%).

**Table 3:** Confidentiality in the consulting room, n=100

**Question: Are you comfortable with the following groups knowing your medical information?**	**Yes**	**No**
Q 5. …all of your ‘party’ (attendants)	55	45
Q 6. …nurses and helpers	81	19
Q 7. …other patients in GOPD	53	47

There were several significant differences in responses according to demographics.

More females than males did not want other patients to know their medical information (57.6%, 31.7% chi-square test, p=0.011). Younger patients had more concerns about confidentiality. More patients aged from 16–25 than those over 35 did not want their attendants to know their medical information (56.1%, 23.1%, p=0.027). The difference was similar concerning other patients knowing but just failed to reach significance (58.5%, 30.8%, p=0.08). In contrast more patients aged over 35 than those aged 16–25 did not want nurses and other helpers to know their information (34.6%, 17.1%, p=0.04) The main reason for not wanting their attendants to know their information was reported as ‘feeling shy’ (29 patients). 12 patients said that information ‘should be private’.

49% of patients did not want their medical information to be made available to other official people outside the consultation (q8, e.g. employer, police).

Patients were asked if there were some specific situation or illnesses where they wanted to see the doctor by themselves (q9). 18% said yes, citing abdominal pain or ‘general check-up’ as the most common situations. Females were more likely than males to want to be seen on their own for some specific illnesses (25.4%, 7.3%, p=0.02). Patients were also asked if they had ever not told the doctor information because they thought it would not be kept private (q10). Only 5% said that they had withheld information.

The overall satisfaction rate with privacy in the department was stated to be 99%.

The final question invited general comments and 78 patients responded: 67 people reiterated the place of privacy, *“privacy is very important during a medical consultation”;* 5 people stated there should be “*one room for one patient in a consultation”;* 3 people thought *“it is important to reduce overcrowding”;* 5 people felt that *“privacy is not important in consultations”.*

## Discussion

This study was designed to discover what patients thought about privacy and confidentiality in this hospital outpatients setting. The results suggest that privacy is a big concern for people in this setting where privacy is often not available. Detailed comparison with previous studies from South Asia and the Middle East is not possible but similar concerns were reported in those settings.

There was consistency between the two key privacy questions - q4 and q11. It is striking that more than half of the patients wanted one consultation per room, given the usual conditions in Nepalese consultations described above. Despite this, only 18% cited specific situations where they would want to consult privately. Patients may have been reluctant to specify the areas of concern – mostly citing ‘abdominal’ conditions. This might also be a product of unfamiliarity with doing surveys. A small number of patients (5%) reported having withheld information due to privacy concerns. The strong, consistent responses to q4 and q11 suggest that these reflect ‘real’ preferences.

Patients were discriminating in answering questions about confidentiality of information. Most (81%) were happy for nurses and helpers to know their information but they didn’t differentiate significantly between their own attendants (55%) and other patients (53%) in terms of confidentiality. This is a surprising result in the Nepalese context, given the huge role played by patients’ attendants in medical care. In addition, around half of the patients (49%) did not want information released to ‘official’ people. These findings further indicate that attitudes of Nepalese patients differ from what has been assumed previously.

Males were less concerned than females with other patients knowing their medical details (68.3%, 42.4%, p=0.01). There was a general tendency for younger and female patients to be more concerned with confidentiality. Previously cited papers from western settings suggest possible reasons for this. Younger people may have been concerned that information about drug, alcohol and sexual issues be kept from their families. Female patients may have been more embarrassed about strangers knowing intimate medical details.

Patients did not offer detailed comments when given the opportunity. However, the remarks that they made supported the quantitative data, underlining the evidence that patients in this context are concerned with confidentiality and privacy.

### Limitations

There are limitations to the study. Patients were unfamiliar with surveys and low literacy levels necessitated that surveys be administered orally. There was only one interviewer, a junior male doctor. It is not clear if this would skew responses and in which direction, however patients may have been less likely to criticize a department in which he was seen as an authority figure. The phenomenon of ‘courtesy bias’ – subjects tending to agree with interviewer’s statements – has been much discussed in relation to research in Asia [20]. This should be considered here although there was agreement between reverse-worded questions (q4, q11). It is likely to have influenced the very high rate of satisfaction with privacy (99%) given the results in all other questions. There was a relatively low number of attendants accompanying the patients in this sample (mean 1.1). The sample may have contained a greater number of ‘independent’ individuals than is the norm in this context.

A convenience sampling method was used and this increased the likelihood that patients had seen the researcher/doctor previously or had chosen to see him. This selection bias may have affected the results in at least two ways. Patients may have been more concerned with privacy in seeing their chosen doctor. They may also have felt less free to express critical opinions.

### Implications

Further research is required to determine the validity of these results. There are implications for the planning of health services in Nepal. The preferences of patients for privacy and confidentiality should be acknowledged in the design and staffing of facilities. There are significant constraints imposed by lack of resources and high patient numbers. However, these results challenge the assumption that Nepalese patients are comfortable with the public nature of their medical care. The implication is that provision should be made for private consultations wherever possible.

These issues should be raised as medical education evolves in Nepal and other parts of South Asia. Western teaching about patient-centred communication is being more widely taught, especially in departments of general practice and psychiatry. This is difficult to implement where the design of facilities and high patient numbers make physical privacy hard to achieve. These issues are very dependent on cultural factors so research needs to be conducted locally to discover what is appropriate now and in the future.

## Appendix

1. How many people have come with you today to see the doctor?

Number of people: ______.

2. Who are they?

a. Parent,

b. Son/daughter, brother/sister,

c. Friend, etc___________.

3. Do you prefer them all to be with you in the consultation?

a. Yes

b. No

c. If not, why not?

4. Are you comfortable with having other patients in the same room as you, consulting with other doctors?

a. Yes

b. No

5. Are you comfortable with all of your party knowing the medical information in your consultation?

a. Yes

b. No

c. If not, why not?

6. Are you comfortable with having nurses and helpers knowing your medical information?

a. Yes

b. No

7. Are you comfortable with other patients in GOPD knowing your medical information?

a. Yes

b. No

8. Do you mind if your medical information is available to other official people

a. Employer? Y/N

b. Insurance companies Y /N

c. Police? Y/N

9. Are there some illnesses or problems where, if you had them, you would prefer to see the doctor by yourself?

a. Yes

b. No

c. If so, what they are?

10. Have you ever not told information to a doctor because you thought that information would not be kept private?

a. Yes

b. No

c. If so, what was the situation?

11. Would you prefer if there was only one doctor in each consulting room?

a. Yes

b. No

12. Are you satisfied with the level of privacy in this outpatients department?

a. Yes

b. No

13. Do you have any other comments about this subject?

a. No

b. Yes

c. If yes, comment:

## Abbreviations

GOPD: General out patients department; BPKIHS: B.P.Koirala Institute of Health Sciences.

## Competing interests

The authors declare that they have no competing interests.

## Authors’ contributions

MM made substantial contributions to the study conception and design and interpretation of results; the drafting and revision of the manuscript; and gave approval to the final version. RC made substantial contributions to the study conception, acquisition of data and the interpretation of results; the drafting and revision of the manuscript; and gave approval to the final version.
